# From “Community of Practice” to “Knowledge Building Community”—A qualitative study of project ECHO as facilitator of adaptive expertise in frontline community workers

**DOI:** 10.1002/lrh2.70028

**Published:** 2025-07-27

**Authors:** Deanna Chaukos, Sandalia Genus, Tim Guimond, Maria Mylopoulos

**Affiliations:** ^1^ Department of Psychiatry Temerty Faculty of Medicine Toronto Ontario Canada; ^2^ Department of Anthropology University of Toronto Toronto Ontario Canada; ^3^ Department of Pediatrics Temerty Faculty of Medicine Toronto Ontario Canada

**Keywords:** adaptive expertise, HIV psychiatry, knowledge building community

## Abstract

**Background:**

Health care is fragmented, stigmatizing, and often does not meet the needs of people living with HIV who present to care with significant complexity. Integrated care is an evidence‐based solution, but rarely is enacted across hospital and community settings. Education for community workers that builds capacity toward integrated care is an essential missing piece.

**Methods:**

Here we describe a qualitative study of the ECHO HIV Psychiatry, a virtual educational series that supports a community of practice of community workers in the HIV sector in Toronto, Canada. The educational series is 9 sessions long and occurs twice/year, reporting here on 4 cycles of the series, from April 2023 to December 2024. Utilizing participant interviews (*n* = 29) and ethnographic observation of education sessions, we conducted an abductive analysis, utilizing concepts of adaptive expertise and Knowledge Building Communities (KBCs) to better understand our participant narratives. Adaptive expertise is a theoretical framework in health professions education that describes capabilities that support healthcare workers to navigate complexity in modern healthcare. KBCs in healthcare leverage collaboration and diverse perspectives to support the generation of new solutions.

**Results:**

Participants' main learning from the ECHO was an *approach* to caring for clients with significant complexity (including mental health concerns), and the learning mechanisms which supported this include: (1) Explicit value placed on diverse domains of knowledge created psychological safety for risk taking; (2) Perspective exchange with people in different roles facilitated confidence for community workers, as well as epistemic humility (humility about what is known or knowable); and (3) Learning in the ECHO led to new knowledge creation through collaboration and improvisation.

**Conclusions:**

Results of this study demonstrate how education can support community workers with an approach to complexity, and that this kind of learning may empower community workers to expand the scope of their role, collaborate across hospital and community, and create new solutions to difficult‐to‐solve problems in health care. These are features of a Knowledge Building Community.

## 1 INTRODUCTION

Health care is fragmented and often does not meet the needs of people living with or at risk for HIV (PLWH) who have mental health challenges.^1^, ^2^, ^3^ PLWH present to care with added complexity, including trauma, adverse childhood events (ACEs), mental health concerns, and medical comorbidity, as well as intersectional social and structural determinants of health. In a siloed healthcare system designed to address one problem at once, PLWH often fall through the cracks; due to structural exclusion and stigma, they do not receive adequate services and care across hospital and community settings.^2^, ^4^, ^5^, ^6^, ^7^, ^8^, ^9^ Integrated care, also known as collaborative or team‐based care, is often proposed as an evidence‐based solution for complex populations.^10^, ^11^, ^12^

However, for PLWH with mental health concerns, there is little access to truly integrated care because services remain fragmented, and most applications of integrated care do not include community workers or peer workers with lived experience.^13^ Community workers in the HIV sector are those who work at social service agencies or at community‐based clinics or testing sites and can include peer workers, case managers, case workers, social workers, public health or outreach nurses, and more. There is little training for providers to support integration of care across hospital and community.^14^, ^15^, ^16^, ^17^ Education for healthcare workers, community workers, and peers that builds capacity by supporting integration and the ability to traverse silos is an essential missing piece to improving care for the most complex patients, including those with HIV.^16^, ^18^ This knowledge gap is described in the literature: specifically, that community‐based education is rare, and existing studies have focused on professional school students rather than community workers.^19^, ^20^ Further, there is a need for education that specifically targets the different and often shared workflow of social service and healthcare providers in the community.^21^

Adaptive expertise is a theoretical framework in health professions education that describes a set of capabilities that support healthcare workers to work nimbly in a complex system—specifically to apply efficient solutions to simple problems, and to adapt knowledge to changing contexts when appropriate.^22^ Adaptive expertise leverages specific learning mechanisms (Table 1)^23^, ^24^ to support and develop the capacity for generating flexible and creative solutions to uncertain or ambiguous problems in health care. Our past research has applied an adaptive expertise pedagogical framework to education initiatives^13^, ^16^, ^17^, ^22^ and has illuminated some of the micro‐, meso‐, and macro‐level barriers to integrated care for PLWH.^25^ Education programs can support integrated care at a micro (clinical) level, through: (1) capacity building for frontline workers; (2) collaboration that supports true perspective exchange (not simply task sharing) between hospital and community workers; and (3) the development of shared language toward integration of both hospital and community expertise for patient care. In a single clinic, this type of education has been shown to support patient‐centered and trauma‐informed care for patients with HIV and mental illness.^13^ Additionally, such educational programs have been effectively scaled to expand reach through Extension for Community Healthcare Outcomes (ECHO) HIV Psychiatry.^26^

**TABLE 1:** Learning mechanisms that support adaptive expertise.

Learning mechanism	Definition
Productive failure	Learners are encouraged to struggle and make mistakes to support optimal learning, prior to receiving direct instruction. Struggle primes the learner for “a time for telling,” where they are most receptive to learning from instruction.
Knowledge integration	Knowledge is best learned when procedural knowledge is tied to conceptual understanding (i.e., the *why* is learned simultaneous to the *what*). This supports conceptual mental models that can integrate diverse kinds of knowledge (for example, biomedical, values, culture).
Meaningful variation	A learner will be better able to apply and adapt knowledge to appropriate contexts when they can understand the implications of variation in a setting. Variation must support meaningful understanding of a concept, rather than simply volume of a repository.
Epistemic humility	Awareness of the limits of one's knowledge, or what is knowable. This stance helps to support psychological safety for productive struggle.
Perspective exchange	Increasingly health care workers are required to integrate diverse kinds of knowledge (biomedical, psychologic, social, cultural, values, etc.) beyond the scope or expertise of their own role. Collaboration that supports integration of diverse perspectives, and not simply task sharing, supports healthcare workers to meet the needs of complex patients.

## 2 RESEARCH INTEREST

Here we report on a qualitative study of the ECHO HIV Psychiatry, specifically exploring if an established Community of Practice (COP)^26^ can support the acquisition of adaptive expert capabilities in participants. We utilize abductive thematic analysis^27^: in response to early data that showed increased generativity and engagement in collective inquiry amongst participants, we utilized the concept of Knowledge Building Communities (KBCs) and adaptive expertise to better understand our participant narratives. KBCs move beyond sharing best practices to purposefully inhabit spaces of complexity, ambiguity, and novelty, where straightforward transmission of knowledge is inadequate.^28^ As such, they have the potential to contribute to a Learning Health System (LHS) approach to health system improvement by supporting opportunities for collective inquiry, intentional learning, and knowledge creation for healthcare workers.

## 3 METHODS

### 3.1 Description of the educational intervention

ECHO (Extension for Community Healthcare Outcomes) is an evidence‐based and innovative distance education model that brings together an expert interdisciplinary team (the “hub”) and primary or community care sites (the “spokes”).^29^, ^30^, ^31^ ECHO HIV Psychiatry was developed to support community workers to develop integrative and mental health competencies to better serve PLWH and emphasizes brief didactic learning and case‐based discussion. To date, 4 cycles of the 9‐session ECHO have occurred, from April 2023 to December 2024, with two cycles recurring annually. Further description of ECHO HIV Psychiatry, including how an adaptive expertise pedagogical framework guided its development, is described previously.^26^

### 3.2 Data collection and analysis

Research ethics approval was granted by the Research Ethics Board at Sinai Health in Toronto, Canada. Participants were recruited to the study from April 2023 to December 2024. Participants were provided information about their participation in the study (interviews and observations of ECHO sessions) and notified that consent for observation was implied in their continued participation. Written consent via online survey was obtained for interviews.

Participant observation was conducted throughout ECHO sessions. Qualitative interviews were conducted at the end of the 9‐session cycle. Delayed participant interviews were also conducted 3–6 months after participation in the ECHO to determine if participant perspectives on learning changed when more time to apply learning had occurred. All participants were invited to be interviewed to ensure diversity of perspectives represented (kinds of roles, agencies, and geographic location of practice). Observational fieldnotes taken by SG and DC focused on engagement and participation; learning and integration; as well as facilitators of learning (what supported or hindered participation, what contributed to psychological safety). Observations were not recorded. Semi‐structured interviews conducted by SG explored the kinds of work participants did at their agency, work challenges, and how they approached these challenges, and what/if any impact the ECHO had on their work/practice. Interviews were recorded and professionally transcribed.

Data analysis occurred from April 2023 to December 2024. Preliminary thematic analysis of the first cycle of the ECHO demonstrated the transformative potential of the COP, specifically that psychological safety facilitated perspective exchange and supported collaborative reformulation of problems and collective solution‐finding.^26^ Our initial work had conceptualized the ECHO as a COP; however, emerging themes showed clear generativity of the group toward new knowledge creation, which was more consistent with the concept of a KBC. Thus, we engaged an abductive approach to better understand participant narratives in our data, guided by concepts of adaptive expertise and KBCs. Using an abductive thematic analytical framework^27^ and the software NVivo (version 12, QSR International Pty Ltd., Victoria, Australia), interview data were iteratively coded by generating open codes, which were then grouped into categories and subcategories to capture identified themes.^32^ Thematic analysis of the interview transcripts was conducted iteratively by SG. The developing themes were regularly reviewed and refined collaboratively with DC and MM, enhancing analytical depth. Fieldnotes taken during observations were utilized to refine and triangulate themes evident in interviews and to explore possible mechanisms of learning more deeply in interviews. Different cohorts of ECHO cycle participants were compared. Codes were interpreted and reconciled through consensus discussion. Additional interviews were pursued until sufficiency was achieved. Sufficiency was achieved with saturation of themes emerging from the data and also with rigor and richness of the data generated.^33^ An audit trail was maintained throughout the process, leading to the development of an analytical framework that focuses on the themes described below.

## 4 RESULTS

Four cycles of ECHO were completed, including 30–40 participants per cycle, with greater than 80% attendance throughout the 9‐week series. It was common for participants to return to ECHO for future cycles, 4–6 per cycle. The majority of attendees worked in nonclinical community roles (case worker, case manager, peer worker).

Semi‐structured interviews with 29 ECHO participants were conducted, including 9 interviews 3–6 months after ECHO participation. Participants represented diverse roles in the HIV sector in the community, including peer or lived‐experience roles, case worker and case manager roles, team leader, and community outreach clinical roles; as well as from different agencies and AIDS Service Organizations focused on serving diverse subgroups with HIV; as well as from different geographic regions in Ontario, including urban and less resourced areas. Thematic analysis demonstrated that participants' main learning from the ECHO was an approach to caring for clients with significant complexity (including mental health concerns) and those who present with difficult‐to‐solve problems. When participants described this newly learned approach, most described a combination of newly acquired or newly applied content knowledge, in combination with a new, more flexible stance toward collaboration and psychiatric complexity. Below we describe the specific learning mechanisms participants' described, which elaborate on themes introduced in earlier work, and how this translates to subsequent, longitudinal on‐the‐job learning. Consistent across examples of applied knowledge was participants ability to describe simultaneous problem‐defining and problem‐solving, which reflects improvisation characteristic of an adaptive expert.

### 4.1 Themes

#### 4.1.1 Explicit valuing of all kinds of knowledge creates psychological safety for risk taking

In a learning environment where diverse kinds of knowledge were valued, participants felt psychologically safe to share their perspective. Hierarchies in medicine have historically prioritized biomedical knowledge. Thus, engagement in perspective exchange was perceived as taking a risk in the interdisciplinary environment, and one that had notably positive consequences in the ECHO (i.e., this led to learning, receiving support, or improved care outcomes for clients). Participants in different roles, including peer roles, felt invited to share their perspectives in the group, especially when invited by people in roles with more traditional power (like psychiatrists). By speaking up, participants felt valued by the group for their expertise and their perspective, and were validated to take further risks.

I feel like that message [importance of a biopsychosocial model] … was relayed multiple times by a different psychiatrist, different folks there… it's so very important to have this biopsychosocial perspective about what is going on, and asking questions, and not assuming, especially when it comes to psychiatric stuff… Now, what is going on, let's sit and talk to people, let's ask questions and be curious, instead of assuming we know exactly what is happening…

Risk taking in the learning environment manifested as inviting uncertainty (saying “I don't know”), challenging a different perspective or expressing an incongruent view, or asking for help. The main facilitators of perspective exchange were recognition that their perspective was of interest to the group, that they had something to offer others, and the belief that doing so would make a difference for client care.

[W]hen I see psychiatrists who work differently, who involve community, which was new to me because of the power they hold, I've never seen psychiatrists ever involve community. If I call a psychiatrist, I've gotten consent from my client and I call the psychiatrist to say the client said that the medication is not working…, you feel it's none of your business and you don't have the qualification to question them about it. Do you know what I mean? So, listening to the psychiatrist that was on the ECHO and hearing how much they respected and listened to community, and supported and validated, it was new, totally new to me, a new experience in a good way.

An additional impact of a psychologically safe learning environment was the opportunity to challenge implicit biases (about psychiatry, or other assumptions made when seen through a singular lens) and to identify impacts of stigma (including on an individual practitioner's practice).

They made me more open to psychiatry in general… Previously, my practice was rooted in abolition and … the harms that psychiatry has caused. But because of that, I didn't engage with it a lot – period. And I think the biggest shift for me was [being] part of those ECHO sessions and seeing it broken down in that way. Also, then from there, hearing from clients at work or speaking with friends that also work in this field or similar fields.

#### 4.1.2 Perspective exchange facilitates both confidence and epistemic humility

Perspective exchange led to increased self‐report of confidence and improved self‐efficacy of community workers. By engaging in perspective exchange with participants in different roles, there was increased awareness of shared challenges. Despite facing difficult‐to‐solve problems in their work, frontline workers described increased confidence in their unique skill sets (often acquired through on the job experiences versus formal education or training). This self‐reported confidence persisted even in the face of greater uncertainty or difficult‐to‐solve problems.

The ECHO [hub] members are very encouraging for community members to speak up. And I think there are many times in … the series where ECHO members were giving positive feedback to a question or a comment like, oh, that's really a great suggestion, I hadn't thought about it that way. That kind of stuff. Again, [this] builds participants' confidence in doing the work they're doing. So yeah, I think it has been really positive…, which is friggin’ amazing that we can do that, because ultimately, it's going to mean that the client is going to get a better experience. They're going to be dealt with in a more effective way, and there are going to be better outcomes for the clients, which is ultimately why we're all here. It's why I'm still here…

For some, the recognition that different roles with different kinds of expertise find the same problems challenging provided greater security in one's skills, what one has to offer in their role, and willingness to improvise (i.e., working across siloes, and even expand the scope of their role).

I think really trying to step back from that tunnel vision of, oh I know the answer, but just kind of … what else might be going on here? Which I've always tried to maintain, but really this ECHO reinforced that, just this wider perspective, what's going on here. Being more curious than assuming I have the answers, asking questions… I can think of a client right now who I consulted with the doctor and a nurse yesterday… Sometimes they don't have the answers, but just being curious, and knowing that the answer could be out there, and not one that we're even thinking of.

Interestingly, this realization—that hard problems were hard for everyone—elicited epistemic humility, that is, the willingness to invite uncertainty and acknowledge the limits of our current knowledge.^34^ In this way, epistemic humility and perspective exchange were mutually mirrored: hearing others' perspectives helped articulate and increase awareness of the limits of one's own knowledge, and inviting uncertainty also amplified engagement in perspective exchange, which supported confidence building. With increased confidence, there was also increased curiosity about others' roles, not simply clinical roles like the MDs, but also people with lived experience and peer workers. There was an interesting co‐occurrence of increased confidence in one's abilities, epistemic humility, and willingness to improvise (expand one's scope), that is suggestive of adaptive expert ability.

I've been working in this field now for six years and it has never felt like, oh I got it, if that makes sense. It's always like there's something new to learn or there's always something that I don't know… And I think that being part of the ECHO sessions and being in the … larger space with people and hearing [the] conversations…, their insight, and the work that they do. It made me feel more confident in the work that I do as well, and I feel like I'm surrounded by peers instead of just people that I'm seeking guidance from. I'm also able to contribute, which I felt was very meaningful and re‐affirming. And then after that, even in the workplace as well, it helped me feel more structured in a way, [in] the work that I'm doing, because it's so self‐directed, a lot of it. Sometimes it's easy to only focus on the client‐specific part and not the program part, but it helped me find meaning in all of it. And even when I [am] interacting with clients about programs, making sure to ask for more insight on what would you like to see from us coming in, outside of just our advisory committee. And realising if it's possible, where can I be engaging clients in the programs and resources that we make, at every step, and not just for them to give feedback but also for me to be able to draw those lines of their importance and how they're all connected.

#### 4.1.3 Learning leads to new knowledge creation/improvisation

Through engagement with the ECHO, participants describe new willingness to integrate and improvise new solutions, including in collaboration with others, on behalf of their clients. Collaborative reformulation (integration of different perspectives toward a new explanation or understanding) was evident through learning new language to describe similar or overlapping phenomena, by integrating learning from one discipline into another, and also by creating new informal teams in the community.

Participants described how learning psychiatric language allowed them to better understand client community files and how to utilize clinical language as a tool for expanding their efficacy and utility in their community role.

I really appreciated them because they were very approachable while also having a lot of insight that was very specific to a lot of the populations that we work with. So, it was useful knowledge and … [s]pecifically, the [session] that was [on] CBT, because while I'm not a trained practitioner, … the goal‐setting thing is helpful for bringing into [work with clients], to building up life skills and independence and helping with that routine. I regularly speak with clients on a weekly basis, so I always go back to the notes that I've written from our last call and see, okay we spoke about these things, make sure to bring up this one next time. Or follow up…, we spoke about this, have you been incorporating that practice over the last week? Which has been beneficial because then it adds more structure to the calls too…

ECHO participants talked about integrating content knowledge into their daily work, and moreover, utilizing that content knowledge to hold ambiguous or contentious parts of their work, or to make breakthroughs with clients where they felt stuck.

The person [from the] case study, they're Indigenous, and so [the ECHO session] did lead to us having a conversation … about some of their trauma. I … [previously] had this mindset of, I'm not going to ask people specifically about their traumas, because I know that … a lot of our folks are connected to different services, so in order to get services, they almost have to retell their story and rehash a lot of things…. I don't think it's always necessary for people to exploit their trauma just for services. But it obviously is the root of some of the substance use and some of the other struggles. And so, I think [the trauma session] allowed me to ask, when appropriate, … it opened the door and for me to feel comfortable. And then, … now that we've talked about it, we can … have a follow‐up conversation. And it did, … the person then [told me] “I don't know what Band I'm from, I don't know this, I don't know that, … can you help me figure this out?” And I'm like, actually, I can, because I have all these resources. So, it was actually really helpful and it opened a door and it's provided us now with something to … figure out, how to provide connection and meaning in [this part of their] life. We still have to tackle some big things, like finances, like housing, all those big things, those don't ever go away, but it's now given us another depth to our interactions, where we can say, okay, let's focus on connection and, you know, history seeking even. So, I really liked that, that was really helpful.

Participants described how the collaborative learning environment supported or inspired participants to create informal teams of their own in the community. For example, some realized that they could recruit help from other organizations and workers to make sure that clients with challenges had multiple resources. Participants began conceptualizing their role in smooth transitions of care, and how the expansion of their scope supported this. Many participants described creative ways they had applied learning from the ECHO to unconventionally solve problems in their work: for example, through trying out a new strategy in direct case management, or by developing new programs or implementing case conferences at their agency. Many participants reflected on peer (PLWH) involvement in the ECHO and wanting to recreate a positive culture for peers in their organization. In this way, the COP shifted into a KBC—where participants collaborated to generate new knowledge and new solutions to existing problems.

Well, with this particular patient, … I was able to say to him, I'd like to refer you to the psychiatrist, but since you and I meet most often, I'd like to be part of the conversation so that I can learn at the same time, and so that we're not reliant on just having a psychiatrist to be able to make good choices for you. Because I think for a lot of people, especially if they've had lots of mental health involvement in their life, and psych involvement, they, in my experience anyway, they tend to be a bit hesitant. They don't really want a new person digging around in their brain and prying. And so, the concept of having me there as well so that I can learn and try and grow as well, I think was helpful, also a bit validating for the patient that I'm being quite candid, like, I don't know everything about this particular topic but I want to do right by you. I think that was helpful.

## 5 DISCUSSION

This study describes how a COP that engages healthcare providers in diverse roles from hospital and community, built on psychological safety, has the potential to support front‐line workers to engage in perspective exchange (across power hierarchies), build confidence, take risks that facilitate learning, integrate new knowledge, and improvise new solutions in collaboration with others. These are foundational elements of a KBC, and our work elucidates how a COP can engage learning mechanisms that support adaptive expertise to become a KBC, impacting care at an individual and community level. Differences between COPs and KBCs are described in Table 2.

**TABLE 2:** Contrasting features of community of practice versus a knowledge building community.

	Community of practice	Knowledge building community
Focus	Shared interest Generating answers	Collective inquiry, that is, generating questions To adapt or improve existing knowledge toward new solutions
Process	Sharing of best practices by experts	Sharing and collaboratively building ‘improvable ideas’ Engaging critically with authoritative sources
Values	Knowledge sharing Shared understanding	Idea diversity—diverse kinds of knowledge are equally valuable (perspective exchange, not simply task sharing) Epistemic humility—“all ideas are improvable” Collective responsibility—“we are better together”

KBCs are characterized by their engagement in collective inquiry that can support individuals to engage in the collective generation of new knowledge in response to practice challenges.^28^ Principles supporting KBCs include idea diversity as well as shared belief that all ideas are improvable.^35^ Our study demonstrates a connection between epistemic humility and the ability to hold one's knowledge lightly, while engaging with new ideas. Modeled epistemic humility—seeing experts, especially those with traditional power, invite uncertainty and engage in perspective exchange—invites others to do so and allows for the dialogue and improvisation necessary to improve ideas. Idea diversity was achieved in the ECHO when participants in various roles and with different life experiences engaged in perspective exchange. Perspective exchange led to increased awareness of the particular expertise of community workers, increasing their confidence. This is especially important for community workers who are not from a professional training pathway, and thus were less likely to have a professional identity based on schooling. Rather, their work identity was influenced by real‐world practice experiences, many without opportunity to reflect in collaboration with others.

An additional principle supporting KBCs evident in ECHO is the constructive uses of authoritative sources (both respect for and critical analysis of authoritative sources to advance knowledge),^35^ again supported by explicit value placed on all kinds of knowledge. The ECHO created psychological safety for front line workers to dynamically engage with psychiatry as a discipline, including with a critical lens. This discourse was importantly influenced by epistemic humility on behalf of the psychiatrists (regarding potential harms of psychiatric involvement), as well as a willingness to confront stigma and bias on the part of community workers. It allowed for unique instances of problem‐solving, for example, creative solutions for what to do about clients who might benefit from psychiatric care but were unlikely to access it from a hospital due to structural barriers and experiences of compound stigma.

Finally, another notable feature of KBCs observed in our data includes the collective responsibility toward building community knowledge.^35^ Participants described renewed energy for the work, impacting confidence and self‐efficacy, related to feeling both “in it together” and “better together,” as well as noting evident effectiveness of their collaborative efforts. These socio‐cognitive dynamics were present in the ECHO, and beyond, as demonstrated by participant examples of impact on their practice and practice environments.

Limitations of this work include small sample size and the reliance on participant self‐report to assess the impact of ECHO participation with no additional external measures of changes in practice. Additionally, the learning mechanisms identified emerged within the context of strong participant engagement over four cycles. It is possible that future cycles involving different participants may result in variations in both the learning environment and outcomes. However, the transferability of the findings is strengthened by the use of a robust theoretical framework to guide data interpretation.

Learning Health Systems informed by health professions education research have been proposed as a necessary missing step for socially accountable healthcare.^36^ This research, which utilizes evidence‐based education to support a KBC driven by community stakeholder priorities, sheds light on the importance of theory‐informed pedagogy for system improvement. A psychologically safe learning environment guided by adaptive expert pedagogical principles can support individuals and teams toward innovative problem solving that can drive health system improvement.

## CONFLICT OF INTEREST STATEMENT

The authors declare no conflicts of interest.

## Data Availability

The data that support the findings of this study are available on request from the corresponding author. The data are not publicly available due to privacy or ethical restrictions.

## References

[1] Emlet CA , Brennan DJ , Brennenstuhl S , Rueda S , Hart TA , Rourke SB . The impact of HIV‐related stigma on older and younger adults living with HIV disease: does age matter? AIDS Care. 2015;27(4):520‐528. doi:10.1080/09540121.2014.978734 25397643

[2] Noest S , Ludt S , Klingenberg A , et al. Involving patients in detecting quality gaps in a fragmented healthcare system: development of a questionnaire for Patients' Experiences Across Health Care Sectors (PEACS). Int J Qual Health Care. 2014;26(3):240‐249. doi:10.1093/intqhc/mzu044 24758750 PMC4041096

[3] Salamat S , Hegarty P , Patton R . Same clinic, different conceptions: drug users' and healthcare professionals' perceptions of how stigma may affect clinical care. J Appl Soc Psychol. 2019;49(8):534‐545. doi:10.1111/jasp.12602

[4] Brezing C , Ferrara M , Freudenreich O . The syndemic illness of HIV and trauma: implications for a trauma‐informed model of care. Psychosomatics. 2015;56(2):107‐118. doi:10.1016/j.psym.2014.10.006 25597836

[5] Durvasula R , Miller TR . Substance abuse treatment in persons with HIV/AIDS: challenges in managing triple diagnosis. Behav Med. 2014;40(2):43‐52. doi:10.1080/08964289.2013.866540 24274175 PMC3999248

[6] LeGrand S , Reif S , Sullivan K , Murray K , Barlow ML , Whetten K . A review of recent literature on trauma among individuals living with HIV. Curr HIV/AIDS Rep. 2015;12(4):397‐405. doi:10.1007/s11904-015-0288-2 26419376 PMC4837695

[7] Maunder RG , Wiesenfeld L , Rawkins S , Park J . Development of the C4 inventory: a measure of common characteristics that complicate care in outpatient psychiatry. J Comorbidity. 2016;6(2):56‐64. doi:10.15256/joc.2016.6.66 PMC5556446 29090175

[8] Orellana ER , Goldbach J , Rountree MA , Bagwell M . Access to mental health and substance abuse services by people living with HIV/AIDS: the case manager perspective. Health Soc Work. 2015;40(2):e10‐e14. doi:10.1093/hsw/hlv023

[9] Black C , Calhoun A . How biased and carceral responses to persons with mental illness in acute medical care settings constitute iatrogenic harms. AMA J Ethics. 2022;24(8):E781‐E787. doi:10.1001/amajethics.2022.781 35976936

[10] Katon WJ , Seelig M . Population‐based care of depression: team care approaches to improving outcomes. J Occup Environ Med. 2008;50(4):459‐467. doi:10.1097/JOM.0b013e318168efb7 18404019

[11] Katon WJ , Lin EHB , Von Korff M , et al. Collaborative care for patients with depression and chronic illnesses. N Engl J Med. 2010;363(27):2611‐2620. doi:10.1056/NEJMoa1003955 21190455 PMC3312811

[12] Chwastiak L , Vanderlip E , Katon W . Treating complexity: collaborative care for multiple chronic conditions. Int Rev Psychiatry. 2014;26(6):638‐647. doi:10.3109/09540261.2014.969689 25553781

[13] Chaukos D , Genus S , Wiesenfeld L , Maunder R , Mylopoulos M . Improving patient‐centered care for HIV and mental illness: exploring hospital and community integration through education. AIDS Care. 2024;36(2):181‐187. doi:10.1080/09540121.2023.2269408 37856839

[14] Sockalingam S , Chaudhary ZK , Barnett R , Lazor J , Mylopoulos M . Developing a framework of integrated competencies for adaptive expertise in integrated physical and mental health care. Teach Learn Med. 2020;32(2):159‐167. doi:10.1080/10401334.2019.1654387 31482737

[15] Youssef A , Wiljer D , Mylopoulos M , Maunder R , Sockalingam S . “Caring About Me”: a pilot framework to understand patient‐centered care experience in integrated care – a qualitative study. BMJ Open. 2020;10(7):e034970. doi:10.1136/bmjopen-2019-034970 PMC7389773 32718923

[16] Chaukos D , Genus S , Kuchar SB , Wiesenfeld L , Maunder R , Mylopoulos M . “It's complicated”: using education to bridge essential care between hospital and community for complex patients with HIV. Acad Psychiatry. 2022;47(1):35‐42. doi:10.1007/s40596-022-01692-3 35906497 PMC9881525

[17] Chaukos D , Genus S , Maunder R , Mylopoulos M . Preparing future physicians for complexity: a post‐graduate elective in HIV psychiatry. BMC Med Educ. 2023;23(1):269. doi:10.1186/s12909-023-04233-0 37081455 PMC10116745

[18] Zhao QJ , Cupido N , Whitehead CR , Mylopoulos M . What role can education play in integrated care? Lessons from the ECHO (Extensions for Community Health Outcomes) concussion program. J Integr Care. 2022;30(4):373‐385. doi:10.1108/JICA-01-2022-0012

[19] Stubbs C , Schorn MN , Leavell JP , et al. Implementing and evaluating a community‐based, inter‐institutional, interprofessional education pilot programme. J Interprof Care. 2017;31(5):652‐655. doi:10.1080/13561820.2017.1343808 28792263

[20] Hosny S , Kamel MH , El‐Wazir Y , Gilbert J . Integrating interprofessional education in community‐based learning activities: case study. Med Teach. 2013;35(Suppl 1):S68‐S73. doi:10.3109/0142159X.2013.765550 23581899

[21] Park M , Choi E , Jeong M , Seo HJ , Kim J , Seo E . Interprofessional educational needs for shared governance of integrated care. Int J Integr Care. 2024;24:15. doi:10.5334/ijic.7674 PMC11086590 38736721

[22] Mylopoulos M , Woods NN . When I say … adaptive expertise. Med Educ. 2017;51(7):685‐686. 28224707 10.1111/medu.13247

[23] Mylopoulos M , Kulasegaram K , Woods NN . Developing the experts we need: fostering adaptive expertise through education. J Eval Clin Pract. 2018;24(3):674‐677. doi:10.1111/jep.12905 29516651

[24] Mylopoulos M , Steenhof N , Kaushal A , Woods NN . Twelve tips for designing curricula that support the development of adaptive expertise. Med Teach. 2018;40(8):850‐854. 30009648 10.1080/0142159X.2018.1484082

[25] Valentijn PP , Schepman SM , Opheij W , Bruijnzeels MA . Understanding integrated care: a comprehensive conceptual framework based on the integrative functions of primary care. Int J Integr Care. 2013;13:e010. doi:10.5334/ijic.886 23687482 PMC3653278

[26] Chaukos D , Genus S , Guimond T , Mylopoulos M . Integration through education: utilizing project ECHO to mitigate fragmentation and support adaptive expert care in HIV psychiatry. J Integr Care. 2024;32(3):321‐330. doi:10.1108/JICA-03-2024-0012

[27] Thompson J . A guide to abductive thematic analysis. Qual Rep. 2022;27(5):1410‐1421. doi:10.46743/2160-3715/2022.5340

[28] Bereiter C , Scardamalia M . Knowledge building and knowledge creation: one concept, two hills to climb. Knowledge Creation in Education. Springer Singapore; 2014:35‐52.

[29] Agley J , Delong J , Janota A , Carson A , Roberts J , Maupome G . Reflections on project ECHO: qualitative findings from five different ECHO programs. Med Educ Online. 2021;26(1):1936435. doi:10.1080/10872981.2021.1936435 34076567 PMC8174483

[30] Komaromy M , Duhigg D , Metcalf A , et al. Project ECHO (Extension for Community Healthcare Outcomes): a new model for educating primary care providers about treatment of substance use disorders. Subst Abuse. 2016;37(1):20‐24. doi:10.1080/08897077.2015.1129388 PMC4873719 26848803

[31] Sockalingam S , Arena A , Serhal E , Mohri L , Alloo J , Crawford A . Building provincial mental health capacity in primary care: an evaluation of a project ECHO mental health program. Acad Psychiatry. 2018;42(4):451‐457. doi:10.1007/s40596-017-0735-z 28593537

[32] Braun V , Clarke V . Using thematic analysis in psychology. Qual Res Psychol. 2006;3(2):77‐101. doi:10.1191/1478088706qp063oa

[33] LaDonna KA , Artino AR , Balmer DF . Beyond the guise of saturation: rigor and qualitative interview data. J Grad Med Educ. 2021;13(5):607‐611. doi:10.4300/JGME-D-21-00752.1 34721785 PMC8527935

[34] Woodruff JN . Accounting for complexity in medical education: a model of adaptive behaviour in medicine. Med Educ. 2019;53(9):861‐873. doi:10.1111/medu.13905 31106901

[35] Scardamalia M . Collective cognitive responsibility for the advancement of knowledge. In: Smith B , ed. Liberal Education in a Knowledge Society. Open Court; 2002:67‐98.

[36] Wood B , Fitzgerald M , Kendall C , Cameron E . Integrating socially accountable health professional education and learning health systems to transform community health and health systems. Learn Health Syst. 2021;5(3):e10277. doi:10.1002/lrh2.10277 34277943 PMC8278438

